# Losing Control in Social Situations: How the Presence of Others Affects Neural Processes Related to Sense of Agency

**DOI:** 10.1523/ENEURO.0336-17.2018

**Published:** 2018-03-08

**Authors:** Frederike Beyer, Nura Sidarus, Stephen Fleming, Patrick Haggard

**Affiliations:** 1 Institute of Cognitive Neuroscience, University College London, London, UK; 2 Institut Jean Nicod, Département D’Etudes Cognitives, Ecole Normale Supérieure, PSL Research University, Paris, France; 3 Wellcome Centre for Human Neuroimaging, University College London, London, UK; 4 Max Planck UCL Centre for Computational Psychiatry and Ageing Research, University College London, London, UK; 5 School of Advanced Study, University of London, London, UK

**Keywords:** Diffusion of responsibility, fMRI, mentalizing network, sense of agency

## Abstract

Social contexts substantially influence individual behavior, but little is known about how they affect cognitive processes related to voluntary action. Previously, it has been shown that social context reduces participants’ sense of agency over the outcomes of their actions and outcome monitoring. In this fMRI study on human volunteers, we investigated the neural mechanisms by which social context alters sense of agency. Participants made costly actions to stop inflating a balloon before it burst. On “social” trials, another player could act in their stead, but we analyzed only trials in which the other player remained passive. We hypothesized that mentalizing processes during social trials would affect decision-making fluency and lead to a decreased sense of agency. In line with this hypothesis, we found increased activity in the bilateral temporo-parietal junction (TPJ), precuneus, and middle frontal gyrus during social trials compared with nonsocial trials. Activity in the precuneus was, in turn, negatively related to sense of agency at a single-trial level. We further found a double dissociation between TPJ and angular gyrus (AG): activity in the left AG was not sensitive to social context but was negatively related to sense of agency. In contrast, activity in the TPJ was modulated by social context but was not sensitive to sense of agency.

## Visual Abstract

**Figure:**
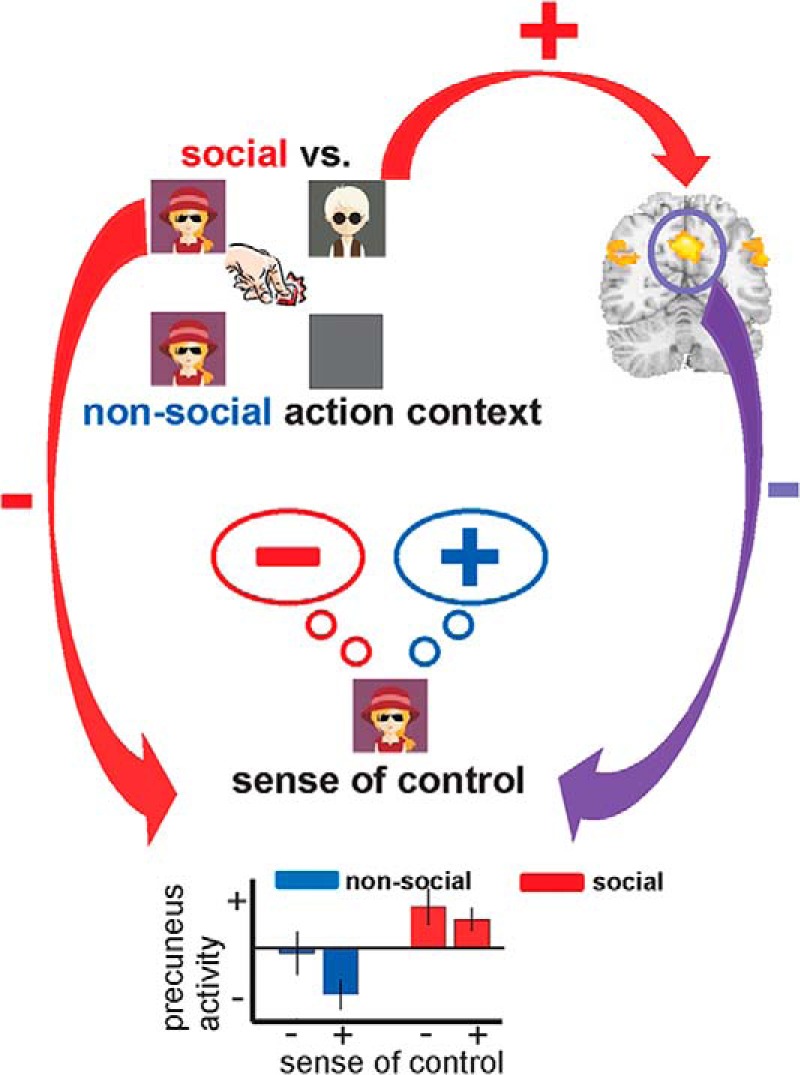

## Significance Statement

The presence of other people can have substantial influence on how individuals act. Understanding the cognitive and neural processes underlying such behavioral changes would allow for better prediction of social context effects on individual actions and, ultimately, support the development of optimized learning environments and interventions aimed at improving social behavior. The work presented here on the neural processes underlying social context effects on sense of agency is an important contribution to research on human cognition, voluntary action, and social psychology.

## Introduction

Cognitive neuroscience research on voluntary action is conducted mostly separately from studies of social behavior, yet a great proportion of human behavior takes place in social contexts. A behavior that is not itself social can nevertheless be influenced by the presence of other agents. For example, diffusion of responsibility is a common phenomenon that describes the effects social context can have on individual behavior: the presence of others decreases the likelihood that someone will interfere in an emergency or social transgression (Darley and Latane, 1968; Chekroun and Brauer, 2002), decreases the effort invested in a work project (Karau and Williams, 1993), increases risk taking (Wallach et al., 1964; Bradley, 1995), and increases aggression (Bandura et al., 1975; Meier and Hinsz, 2004).

However, although social psychology has long acknowledged that social contexts greatly affect the way people act (Asch, 1956; Milgram, 1963; Wagner et al., 1986; Brewer et al., 1993; Bond and Smith, 1996; Blass, 1999; Leach and Spears, 2009), little is known about how the presence of others affects the cognitive processes underlying voluntary action.

Sense of agency—the feeling that we are in control of our actions and their consequences—is closely linked to feelings of responsibility (Moretto et al., 2011; Frith, 2014). A previous study using event-related potentials investigated how diffusion of responsibility influences outcome processing and sense of agency (Beyer et al., 2017). This study employed a task in which participants acted in either a social or nonsocial context but had the same control over action outcomes in both settings. In social trials, participants reported lower sense of agency and showed reduced outcome monitoring, reflected in a reduced feedback-related negativity (FRN).

In parallel, studies have shown that the difficulty of the action selection process plays an important role in sense of agency (Sidarus et al., 2013; Chambon et al., 2014; Sidarus and Haggard, 2016; Sidarus et al., 2017): disruption of action selection by incongruent priming or incongruent flanker stimuli reduces sense of agency. This effect is related to activity in the left angular gyrus (AG), with enhanced AG activity during action selection being correlated with a reduction in sense of agency in high-conflict trials (Chambon et al., 2013). Similarly, increasing working memory load has been found to reduce sense of agency (Hon et al., 2013; Howard et al., 2016; Wen et al., 2016).

**Figure 1.**
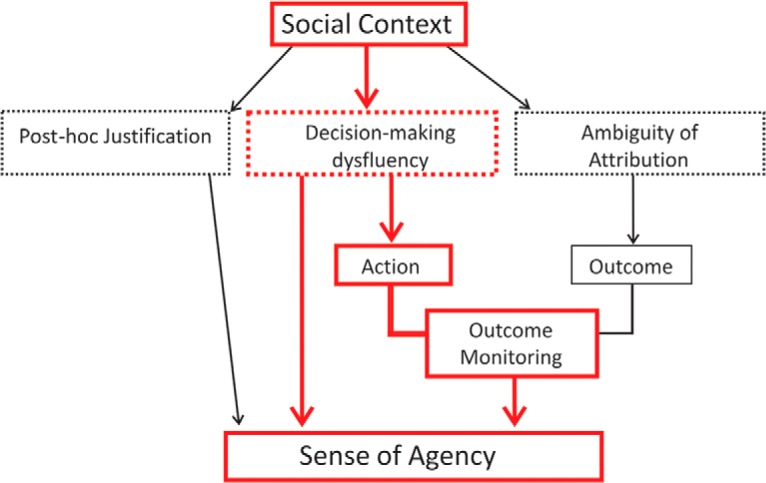
Model of social context influences on sense of agency. The figure shows a previously proposed model (Beyer et al., 2017) of social context influences on sense of agency. It proposes that mentalizing processes increase dysfluency in the action selection phase, reducing sense of agency and outcome monitoring.

An appealing synthesis of these findings is that social contexts reduce sense of agency and the monitoring of action outcomes by making the action selection process less fluent (Fig. 1). In a social context, mentalizing processes may make it more difficult to decide if and when to act, reducing participants’ sense of agency and their monitoring of action outcomes (Beyer et al., 2017).

The present fMRI study investigated the influence of social context on neural processes during the action selection phase of a task and related these to the sense of agency over action outcomes. We adapted the balloon-analog risk-task (BART; Lejuez et al., 2002), in which participants had to stop an inflating balloon from bursting by pressing a button. Although this button-press was costly (resulting in the loss of monetary points), not acting (letting the balloon burst) was more costly. In the social context, another player was present (represented by an avatar on the screen), who could act instead of the participant, but, in the trials analyzed, did not.

We made the following predictions, according to the above model of diffusion of responsibility. (1) During action phases of social trials, we should find increased activity in brain areas associated with mentalizing processes [temporo-parietal junction (TPJ), medial prefrontal cortex (mPFC), precuneus, temporal poles; Lieberman, 2007]. (2) There should be a negative relationship between activity of mentalizing areas during the action phase and sense of agency. (3) In social trials, participants should show reduced outcome monitoring (i.e., reduced reactivity of prefrontal areas to outcome presentation)

If thinking about a coplayer’s intentions reduces sense of agency, participants who are more prone toward adopting someone else’s point of view could be more strongly affected by social contexts. In an exploratory analysis, we tested whether the reduction of sense of agency in social contexts is related to personality factors such as locus of control or the tendency to adopt other people’s perspectives. For these analyses, we included behavioral data and personality questionnaire scores of participants who participated in the same experiment but performed the task outside the fMRI scanner.

## Materials and Methods

### Procedure

Participants were invited into the lab in mixed-sex pairs of two. Participants wishing to perform the task in the MRI scanner and participants who were to perform the task outside the scanner were recruited separately through different study advertisements, and none of the participants attending the study together reported knowing each other. Participants received instructions together and gave informed written consent. They were instructed that they would be playing together, with one participant inside the MRI scanner. One participant was then shown to a computer where they would be performing the task, the other was brought into the MRI. In fact, both participants were playing separately. After the task was finished (and, for the MRI participants, after the acquisition of structural images), participants filled out a postexperimental questionnaire assessing their belief regarding the other player’s participation in the game, as well as personality questionnaires (see below). Participants were then fully debriefed, paid, and thanked for their participation. Participants received £10.00 per hour, plus any earnings from the task.

All procedures were approved by the local ethics committee and were conducted according to the Declaration of Helsinki.

### Participants

Twenty-four participants (all right-handed) participated in the MRI experiment. Data of one participant had to be excluded because of movement of >4 mm during functional scans. Data of two participants had to be excluded because they failed to stop the balloon on the majority of trials, resulting in trial numbers of <10 per experimental condition. Finally, during data analysis, one further participant had to be excluded because lack of variability in their control rating in at least one task run, resulting in failure of the parametric modulation model. Thus, data of 20 participants (7 male; age 18–27 years, mean 22 years) were included in the MRI analyses.

For correlation analyses between diffusion of responsibility scores and personality variables, we additionally included data of 19 participants (9 male, mean 22 years old) who performed the task without MRI. Of the 24 testing sessions, 1 non-MRI participant failed to attend and had to be replaced by a confederate; for 4 participants, the experiment was terminated prematurely due to technical failure, resulting in a total of 19 participants with behavioral but not neural data.

### Task

The task was modeled after the one used in Beyer et al. (2017) and designed to mimic diffusion of responsibility scenarios, in which the presence of another potential agent may motivate a person to not take action. It provided immediate action feedback, such that outcome predictability was identical across social and nonsocial action contexts, as in the social context, there was no ambiguity as to which player caused a given outcome. The payoff structure of the task was designed to mimic the cost–benefit structure of typical bystander scenarios in which bystanders have to weigh the cost of, for example, helping an injured person (e.g., loss of time) against the cost of not helping (e.g., further injury to the victim). As such, actions were designed to be costly, but less costly than not acting.

**Figure 2.**
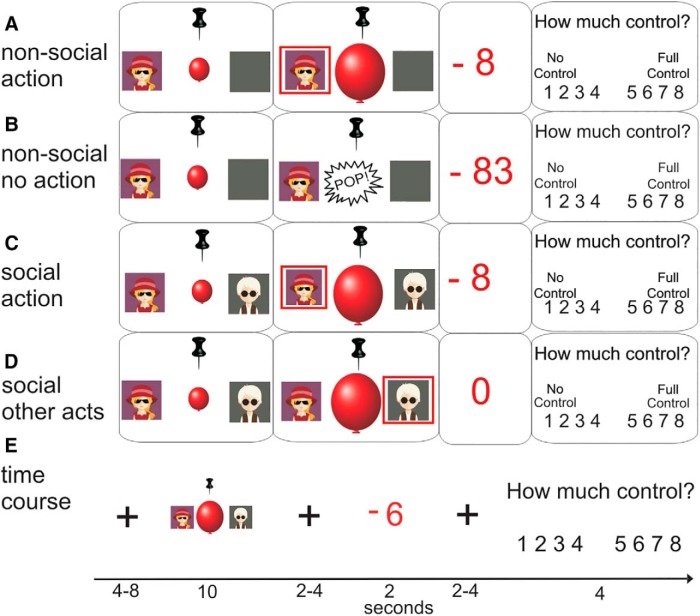
Task design. In nonsocial trials, participants saw their own avatar and an inflating balloon. Successful stopping of the balloon by button press resulted in loss of monetary points depending on balloon size (***A***). If the balloon popped, a large number of points was lost (***B***). In social trials, action–outcome contingencies were identical to those in nonsocial trials (***C***). If the coplayer acted first, the participant lost no points (***D***). The task timing is shown in ***E***.

Inside the MRI, participants used two button boxes with four buttons each to control a balloon-inflation game. They observed the computer screen through a mirror fixed to the head coil. Participants outside the scanner used eight neighboring buttons on a standard computer keyboard. Fig. 2 shows an outline of the task time course as well as the different conditions. During the task, each participant was represented by their own individual avatar (http://www.freepik.com). The task consisted of three runs of 20 trials each (10 nonsocial trials and 10 social trials, in randomized order). Before the first run, participants played a short practice block of two nonsocial trials and two social trials. At the beginning of each trial, participants saw either their avatar alone or their own avatar next to the other player’s avatar. After 2 s, a small balloon appeared in the center of the screen and started inflating at variable speed, which increased unpredictably on each trial. Above the balloon was an image of a pin. As soon as the balloon touched the pin, it would burst and the word ‘pop’ would appear on the screen. At any time, the participant could press a button to stop the balloon. In that case, the balloon stopped and a red rectangle appeared around the participant’s avatar to indicate it had been them who stopped the balloon. Due to the unpredictable change in speed, it was risky to wait to the last possible moment before stopping the balloon. In social trials, if the other player acted before the participant, the balloon would stop and the rectangle would appear around the coplayer’s avatar. The duration of the action phase was fixed to 8 s, with a fixation cross presented at the end if the balloon was stopped or popped earlier.

After a variable interval of 2–4 s, the outcome was presented. To ensure comparability to the previous study using this design (Beyer et al., 2017), actions in this task were costly. If they stopped the balloon, participants lost 1–30 points, depending on the size of the balloon: the bigger the balloon was when stopped, the fewer points they lost; however, the outcome was not entirely predictable from the balloon size. If the balloon burst, participants lost 80–99 points. In social trials, if the coplayer stopped the balloon, participants lost 0 points. If the balloon burst, participants were told that both players would lose 80–99 points. The outcome was presented for 2 s, followed by a jittered interval of 2–4 s. After that, participants saw on the screen the question, “How much control did you feel over the outcome?” Below the question, they saw the numbers 1–8 aligned as a scale with the labels “No control” (left of the 1) and “Complete control” (right of the 8). Participants were instructed that they should rate the amount of control they felt they had had over the number of points they had lost in the given trial. If participants did not make a response within 4 s, the message “Please make a control rating!” was displayed for 2 s and the trial ended (mean number of missed ratings per participant = 0.33; maximum number of missed ratings = 2). Trials were separated by a jittered intertrial interval of 4–8 s.

The coplayer’s behavior was programmed to ensure the participant would act to stop the balloon on the majority of social trials. The coplayer would only stop the balloon if the participant had stopped the balloon on the majority of social trials so far (>50%, that is, for the coplayer to act, the participant must have acted on at least one social trial more than the coplayer), and for a maximum of three trials per block. Thus participants performed individual actions in a context of social uncertainty.

At the beginning of each block, participants received an endowment of 1000 points, which corresponded to 1.00£, from which that block’s losses were subtracted.

### Personality questionnaires

To assess interpersonal variability in relevant personality traits, we used Rotter’s Locus of Control (LoC) scale (Rotter, 1966) and the Interpersonal Reactivity Index (IRI; Davis, 1980).

The LoC contains 29 forced-choice items in which subjects have to choose one of two statements depending on which they agree with better. It was designed to assess subjects’ expectancies for control over reinforcement. Higher scores on this scale represent a more external locus of control, lower scores a more internal locus of control.

The IRI consists of 28 items and 4 subscales: perspective taking, fantasy, empathic concern, and personal distress. Answers are given on a 5-point Likert scale with the labels “Does not describe me well” (1) and “Describes me very well” (5). We were particularly interested in the subscale “perspective taking,” which contains items such as, “I sometimes find it difficult to see things from the ‘other guy’s’ point of view.”

### Behavioral data

Our analyses focused on trials in which the participant, not the coplayer, acted. Each trial was classified according to experimental condition (social vs. nonsocial) and trial outcome (number of points lost). The dependent variable was the agency rating made at the end of the trial.

All trialwise data were entered into a mixed-effects linear model in R (Bates et al., 2014; Team, 2014), with participant as random effect. The advantages of this analysis approach over traditional analyses, such as ANOVA, are its robustness against differences in trial numbers between conditions, the simultaneous analysis of within-subject and between-subject variability, and the possibility to analyze conditions with low trial numbers (Bagiella et al., 2000; Baayen et al., 2008). The experimental factor of social context (coded 0.5 for nonsocial, –0.5 for social) and outcome magnitude (standardized within participants), as well as the interaction between the two, were entered as predictors of agency ratings. These fixed effects were also allowed to vary between participants (i.e., random slopes model). In an additional analysis, we modeled outcome magnitude by experimental condition and reaction time (RT; standardized within participants).

Analyses were conducted using the lme4 package (Bates et al., 2014) in R. Parameter estimates (β) and their associated *t* tests (*t*, *p*), calculated using the Satterthwaite approximation for degrees of freedom (Kuznetsova et al., 2015), are presented to show the magnitude of the effects, with bootstrapped 95% confidence intervals (Efron and Tibshirani, 1994).

For analysis of between-subject variability in sensitivity to social context and personality variables, we calculated for each participant the mean difference in agency ratings between nonsocial and social trials. Again, for this analysis, we considered only trials in which the participant stopped the balloon. We then correlated this diffusion of responsibility score with the locus of control score and the score on the subscale “perspective taking” of the IRI.

Distribution of analyzed datasets and statistical tests performed are given in the Table 1.

**Table 1.** Statistical analysis

Location	Data structure	Statistical test	Confidence interval/achieved power
a	Nonnormal distribution	Mixed-effects linear model	0.12, 0.95
b	Nonnormal distribution	Mixed-effects linear model	–0.73, –0.43
c	Normal distribution	*t* test	0.1, 0.55
d	Normal distribution	*t* test	–0.01, 0.28
e	Normal distribution	*t* test	–0.1, 0.4
f	Normal distribution	*t* test	0.1, 0.7
g	Nonnormal distribution	Mixed-effects linear model	0.18, 0.68
h	Nonnormal distribution	Mixed-effects linear model	–0.10, –0.02
i	Nonnormal distribution	Mixed-effects linear model	–0.16, –0.01

### MRI recording and image preprocessing

MRI images were recorded using a 3.0-Tesla Siemens trio scanner and a 32-channel head coil. T1-weighted structural images were recorded with the following specifications: matrix, 240 × 256; 176 sagittal slices; voxel size, 1 × 1 × 1mm. Three runs of functional images were recorded with the following specifications: matrix, 64 × 64; 48 transversal slices in ascending order; 180 volumes per run; TR, 3.3 s; TE, 30 ms; voxel size, 3 × 3 × 3mm, including a 0.3-mm slice gap.

Preprocessing was conducted using FSL 5.0 (Smith et al., 2004; Jenkinson et al., 2012) to allow for independent component analysis (ICA)-based correction of movement artifacts. Motion correction was performed with MCFLIRT, and images were brain extracted and spatially smoothed with a 5-mm kernel. We then used the toolbox AROMA (Pruim et al., 2015) to perform ICA-based correction for motion artifacts. This approach has been shown to be superior to the standard approach of including motion regressors in first-level analyses (Pruim et al., 2015). Functional data were then normalized into MNI space using the participant’s structural image for coregistration, applying nonlinear registration with 12 degrees of freedom, and high-pass filtering with a cutoff of 100 s.

### MRI statistical analysis

All further MRI analysis was conducted using SPM12 (Ashburner et al., 2014), to allow for the use of SPM-based toolboxes. At the subject level, we defined task regressors for the action phase for social and nonsocial trials. These included only trials in which the participant stopped the balloon, and regressors were modeled from trial onset for the duration of 10 s (2 s of condition information and 8 s of action phase). As parametric regressors for the action phase, we included trialwise agency ratings. For these trials, we also modeled the outcome presentation with a duration of 2 s and included outcome magnitude as a parametric regressor (with higher numbers indicating worse outcomes). Regressors of no interest included the agency rating phase, trials in which the balloon popped, trials in which the coplayer stopped the balloon, and button presses.

The contrast “action phase social” > “action phase nonsocial” was defined at the subject level. For the parametric modulation, we weighted the linear effect of the parametric modulator negatively for both conditions. At the group level, we performed one-sample *t* tests for these contrasts against zero. The same was done with the contrast “outcome social” > “outcome nonsocial.” For whole-brain MRI analyses, we used a significance threshold of *p* < 0.01 family-wise error (FWE) corrected at the cluster level (initial uncorrected threshold *p* < 0.001).

For the parametric analysis, we were specifically interested in whether regions more active during the action phase in social trials would be negatively related to agency ratings across task conditions. This was based on our hypothesis that mentalizing during the action phase should increase action selection dysfluency and thus reduce sense of agency. To obtain regions of interest (ROIs) for testing the parametric modulation for the action phase, we used the toolbox marsbar (Brett et al., 2002) to define ROIs based on the significant clusters from the action phase social > action phase nonsocial comparison. We then tested these ROIs for negative modulation by control ratings with a significance threshold of *p* < 0.05. We also include a more stringent *p*-value corrected for running the tests across 4 ROIs. Because, in SPM, parametric regressors are orthogonalized to the main regressor, this comparison is independent of the main contrast used to define the ROIs. Moreover, we specifically investigated parametric modulations across task conditions; that is, we looked for neural activity related to sense of agency independently of social context. To test for interaction effects in which the parametric modulation by agency ratings differs across social context, we performed a paired *t* test directly comparing the parametric effect between experimental conditions.

## Results

### Behavioral analyses

Our main behavioral analyses focused on the data from the 20 subjects performing the task in combination with fMRI scanning. Comparing task performance between social and nonsocial contexts, we found that participants lost overall fewer points in the social context [mean 26.2 (standard error 0.9)] compared with the nonsocial context [40.3 (0.9); *t*_19_ = 10.2, *p* < 0.001]. This reflected adaptive behavior in the social context, as there were significantly fewer popped balloon trials in the social context [6.0 (0.4)] than the nonsocial context [10.2 (0.3); *t*_19_ = 8.0, *p* < 0.001] and in social context trials, the coplayer stopped the balloon more often [7.3 (0.3)] than it popped [6.0 (0.4); *t*_19_ = 3.5, *p* < 0.01].

**Figure 3.**
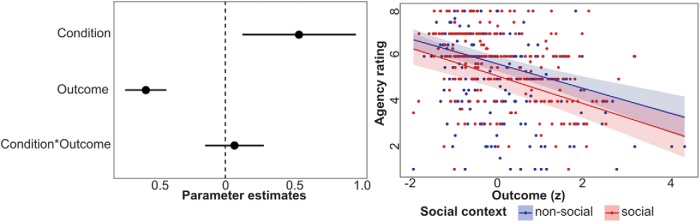
Behavioral results. Participants (*n* = 20) reported lower sense of agency for worse outcomes (higher number of points lost). Independently of outcome magnitude, agency ratings were lower in social compared to nonsocial trials.

The mixed model analysis showed significant main effects of social context (β = 0.54; *t*_19.5_ = 2.55; *p* < 0.05; CI = [0.12, 0.95])^a^ and outcome magnitude (β = –0.58; *t*_18.5_ = 7.58; *p* < 0.001; CI = [–0.73, –0.43])^b^, but no interaction (*p* = 0.54). As can be seen in Fig. 3, participants reported lower sense of agency for worse outcomes, and lower sense of agency in the social context. These results replicate the finding of Beyer et al. (2017), showing that acting in a social context reduces subjective sense of agency, independently of outcome magnitude.

The model for outcome magnitude in trials in which participants stopped the balloon showed that outcomes were related to reaction times (β = 0.24; *t*_22.7_ = 6.06; *p* < 0.001; CI = [0.17, 0.32]), as is in the nature of this task, but not to experimental condition (*p* = 0.14) or their interaction (*p* = 0.16).

In an additional analysis of agency ratings, we included the data from the 19 participants performing the task without MRI. In this analysis, agency ratings were modeled by the between-subject factor of group (with or without MRI) and the within-subject factors social versus nonsocial context and outcome magnitude. This analysis replicated the above findings, with no significant effect for the group factor or its interactions (Table 5).^g^

Mean and distribution values for the personality questionnaires are given in Table 2. None of the personality variables were correlated with diffusion of responsibility scores (defined as difference in mean agency ratings between experimental conditions; all *p* > 0.4). Importantly, mean diffusion of responsibility scores did not differ between participants performing the task inside [0.46 (0.21)] and outside [0.26 (0.14); *p* = 0.43] the MRI.

**Table 2.** Personality questionnaire scores

Value	Mean	SD	Minimum	Maximum
Interpersonal reactivity index				
Perspective taking	18.7	3.5	10	26
Empathic concern	19.2	5.6	8	27
Fantasy	18.0	5.3	6	27
Personal distress	13.0	4.5	2	24
Locus of control	12.0	3.8	5	19

### Neural reactivity to social context

**Figure 4.**
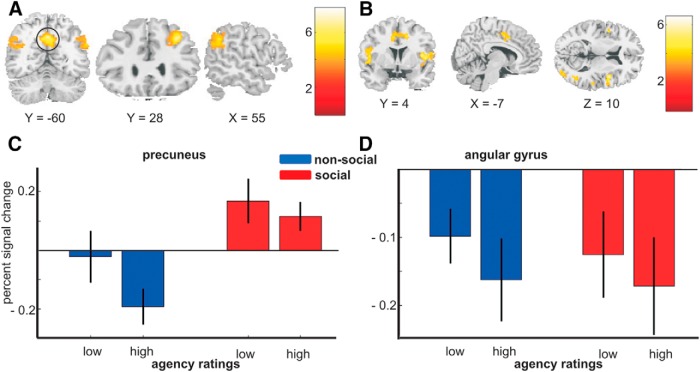
Imaging results for the action phase. ***A***, Increased activity in social trials in precuneus, bilateral temporo-parietal junction, and middle frontal gyrus. ***B***, Increased activity during nonsocial trials was found in anterior cingulate cortex, bilateral insula, and middle occipital cortex, among other regions. Activity in the precuneus (***C***; ROI circled in ***A***) and the left angular gyrus (***D***) was negatively related to sense of agency on a trialwise basis. *n* = 20.

For the action phase of social trials, compared with nonsocial trials, we found increased activity in the bilateral TPJ, the precuneus, and the right middle frontal gyrus (MFG; Fig. 4*A*; Table 3). Both the TPJ and the precuneus are typically found to be activated during tasks that involve mentalizing (Lieberman, 2007; Schurz et al., 2014). At a liberal threshold of *p* < 0.001 uncorrected, we also found a cluster in medial prefrontal cortex (*x* = 4, *y* = 46, *z* = 38).

**Table 3.** Contrast values for the action phase

Region	Peak MNI coordinates (*x*, *y*, *z*)	Cluster peak (*t* value)	Cluster size (*n* voxels)
Social > nonsocial			
Precuneus	–2, –62, 40	7.82	1011
Middle frontal gyrus	30, 26, 40	7.72	358
Right TPJ	44, –54, 22	6.48	676
Left TPJ	–44, –52, 34	4.96	268
Nonsocial > social			
Insula	40, 0, 12	6.91	351
Postcentral gyrus	52, –24, 20	6.11	334
Cingulate gyrus	12, 10, 38	5.98	407
Middle temporal gyrus	52, –56, 2	5.97	473
Middle temporal gyrus	–46, –62, –2	5.41	120
Precentral/inferior frontal gyrus	–50, 2, 10	5.11	186
Middle occipital gyrus	30, –90, 8	4.93	143

The reverse contrast for the action phase, nonsocial > social, showed significant clusters in the right postcentral gyrus, the bilateral insula, the left middle frontal gyrus, the right middle temporal gyrus, the right cingulate gyrus/supplementary motor area (SMA), the left prefrontal/inferior frontal gyrus, and the right middle occipital gyrus (Fig. 4*B*). None of these regions were parametrically related to sense of agency (all *p* > 0.09).

### Neural correlates of sense of agency

Next, we tested whether the increased activity in mentalizing areas observed in the social context was negatively related to sense of agency. ROIs were defined for the clusters of the social > nonsocial contrast (precuneus, MFG, and bilateral TPJ). The contrast values for the parametric modulation by agency ratings were averaged across all voxels in each ROI. This analysis showed significant negative modulation of activity by agency ratings for the precuneus [*t*_19_ = 2.28; *p* = 0.017 (0.068 corrected); Fig. 4*C*].^c^ The parametric modulation approached significance for the MFG [*p* = 0.071 (0.262 corrected)]^d^ but was not significant for the bilateral TPJ [*p* = 0.143 (0.463 corrected)].^e^ That is, activity in the precuneus during the action phase was negatively related to the agency rating made at the end of each trial. To assess whether relationships between agency and activity varied across social context, we compared the parametric modulation between social and nonsocial conditions in a paired *t* test. This showed no significant effect for the precuneus ROI (*p* > 0.4). Thus, trialwise activity in the precuneus showed two independent main effects: activity was higher in social (vs. nonsocial) conditions, and higher for decreased sense of agency.

To test whether precuneus activity mediated social context effects on sense of agency, we performed a mediation analysis as implemented in the Mediation Toolbox (http://wagerlab.colorado.edu/tools) developed by Wager et al. (2008). To get trialwise measures of precuneus activity, we used the BASCO toolbox (Göttlich et al., 2015) to create trialwise β-series for the precuneus ROI. We tested a multilevel mediation model with social context as predictor, precuneus activity as mediator, and agency ratings as dependent variable. This showed significant effects of social context on precuneus activity (*p* < 0.001), precuneus activity on agency ratings (*p* < 0.05), and social context on agency ratings (*p* < 0.05), but no mediation effect (*p* = 0.4).

As described in the Introduction, we hypothesized that decreased agency in our task may be driven by action dysfluency, an effect previously observed by Chambon et al., (2013), who found the left angular gyrus to be negatively related to sense of agency in incompatibly primed trials of a forced-choice instrumental task. To test whether activity in the left AG was similarly related to sense of agency in our task, we defined a 5-mm spherical ROI around the peak coordinates (*x* = –36; *y* = –69; *z* = 45) given in Chambon et al. (2013). This ROI did not overlap with the left TPJ cluster observed in the main social > nonsocial contrast. As predicted, we found a significant negative effect for the left AG for the parametric modulation by agency ratings during the action phase [*t*_19_ = 2.12; *p* = 0.023 (0.090 corrected); Fig. 4*D*].^f^ Activity in the AG was not modulated by experimental condition during the action phase (*p* = 0.21), and the interaction between agency ratings and social versus nonsocial context was not significant for the AG (*p* = 0.36). This result indicates the AG is modulated by agency irrespective of social context.

To allow for better comparison between the results for the different ROIs, we conducted an additional ROI analysis using 5-mm spherical ROIs centered around the cluster peaks for the social > nonsocial comparison contrast. This showed essentially the same results, with a significant effect for the precuneus [*t*_19_ = 2.33, *p* = 0.015 (0.060 corrected)] and no effect for the MFG [*p* = 0.056 (0.207 corrected)] or TPJ [*p* = 0.17 (0.526 corrected)].

Although reaction times are sometimes considered a confound in parametric analyses, in speeded reaction time tasks, sense of agency and reaction times are not necessarily correlated (Chambon et al., 2013). However, in the current study, reaction times were related to outcome magnitude as shown above, which was in turn related to sense of agency. Thus, whereas in speeded reaction time tasks monitoring of reaction times might be used as a proxy for task difficulty, in our task, reaction times were strategically adjusted as a function of the balloon inflation rate and perceived risk. Thus, any link between reaction times and sense of agency is more likely to be indirect and mediated by the outcomes. However, to test whether the effects observed here might be entirely dependent on reaction times, we set up mixed-effects linear models, modeling trialwise activity of precuneus and AG. For each ROI, we ran three models, using both agency ratings and reaction times, using only agency ratings, and using only reaction times as predictors. For both ROIs, the main effect of agency ratings on neural activity remained practically unchanged by inclusion or exclusion of the reaction time factor in the model (Table 5). Thus, we show that the parametric modulations by agency ratings observed here are not driven by effects of reaction times.

For the model of precuneus activity, we found a significant agency × RT interaction effect, with *post hoc* tests showing that agency modulated precuneus activity in trials with average and slow reactions, but not in those with fast reactions (Table 5).

### Neural activity during the outcome phase

The main contrast nonsocial > social showed bilateral activity in occipital cortex reaching into temporal and parietal cortices (Fig. 4*E*; Table 4). The reverse contrast showed activity in mentalizing areas (TPJ, precuneus; Fig. 4*F*; Table 4). There were no significant effects of the parametric modulation by outcome magnitude. The latter finding is somewhat surprising given that striatal reactivity would be expected to correlate with outcome magnitude. However, with increasing task experience, participants may have become fairly good at roughly predicting outcome magnitude. To test this, we performed a parametric analysis focusing on functional run 1 only, showing the expected striatal reactivity to outcome magnitude during early task stages: at a significance level of *p* < 0.001 uncorrected, cluster size >10 voxels, we found a negative relationship between number of points lost and activity in the left putamen, cingulate gyrus, and precentral gyrus (Table 4).

**Table 4.** Contrast values for the outcome presentation

Region	Peak MNI coordinates (*x*, *y*, *z*)	Cluster peak (*t* value)	Cluster size (*n* voxels)
Nonsocial > social			
Middle temporal gyrus	40, –58, 6	6.86	709
Middle occipital gyrus	–40, –80, 0	6.54	536
Cuneus	–20, –86, 28	5.61	346
Precuneus	26, –60, 34	5.55	240
Social > nonsocial			
Middle temporal gyrus	60, –6, –14	6.25	106
Precuneus	–4, –52, 38	6.16	548
Right TPJ	60, –58, 34	6.00	219
Left TPJ	–52, –56, 34	4.59	153
Parametric modulation by outcome magnitude, run 1 only, *p* < 0.001 uncorrected			
Putamen	–22, 12, 2	5.3	53
Cingulate gyrus	–8, –4, 42	4.4	25
Precentral gyrus	–28, –30, 48	5.6	18
Precentral gyrus	–24, –14, 68	5.8	43
Precentral gyrus	–30, –24, 64	4.6	20

## Discussion

We examined the relationship between mentalizing processes while participants acted in social and nonsocial settings and their subjective sense of agency over action outcomes. We replicated previous findings showing reduced sense of agency in the social context. Increased precuneus activity during social trials, an effect typically linked to mentalizing in previous studies, was related in our study to a reduction in the sense of agency over action outcomes. We also found a dissociation between anterior TPJ, which was sensitive to social context but not sense of agency, and posterior AG, which showed the opposite effect.

### Neural correlates of social context

We found increased activity in the bilateral TPJ, precuneus, and right MFG during the action phase of social compared with nonsocial trials. Both the TPJ and the precuneus are associated with mentalizing processes (Lieberman, 2007; Schurz et al., 2014).

Among different mentalizing tasks, the precuneus is most consistently found active in tasks that involve mental imagery, such as visuo-spatial perspective taking, false belief tasks, or trait judgments (Schurz et al., 2014). Our task did not involve any obvious visuo-spatial perspective taking. However, efficient performance might depend on simulating the coplayer’s strategies and level of risk affinity. Thus, simulating another’s possible actions, though not their spatial perspective, was presumably an integral part of the mentalizing evoked during social trials. The precuneus has also been found to show increased activity in agency-related motion tasks, when participants experience increasing mismatch between their movement and visual feedback and attribute motion to another agent (Farrer and Frith, 2002; Nahab et al., 2011). However, this area has generally received less discussion than the angular gyrus or TPJ in the context of agency experiments. For example, previous studies could not clarify whether precuneus activation was action-related or strictly social. In previous experimental designs, precuneus activation could reflect one’s own lack of agency or provide evidence that another person is the agent. Our data give a clear answer to this question: we clearly identify precuneus activation associated with reduced sense of agency. A recent study using retrograde tracing in macaques (Passarelli et al., 2017) suggests that the medial precuneus is involved in higher-level visuospatial cognition and is connected to areas associated with abstract action planning.

The general pattern of activity we found (strong activity in medial precuneus and bilateral TPJ, but not mPFC) is consistent with that described by Schurz et al. (2014) as commonly observed in “rational action” tasks, in which participants have to infer the goal of an agent’s action from nonverbal stimulus material. However, it has to be noted that at a more liberal threshold, we did observe increased mPFC activity in social trials and thus do not base strong conclusions on this null finding.

During the action phase of nonsocial trials, participants showed increased activity in postcentral gyrus, SMA, occipital cortex, insula, and cingulate gyrus. Interestingly, similar results were found by Kuo et al. (2009), who compared deliberative and intuitive social tasks. They found stronger activity in precuneus, inferior parietal areas, and MFG for the deliberative task, which required circular reasoning (each player’s optimal strategy depends on the other’s behavior), and increased activity in insulae and cingulate cortex for the intuitive, relatively low-effort task. Our findings similarly suggest that in nonsocial trials, the balloon task posed a straightforward, intuitive task that evoked strong focus and task engagement. In social trials, this focus was to a degree reduced in favor of mentalizing processes that were necessary to adjust individual strategies to the other’s behavior.

### Social context and the neural basis of sense of agency

Replicating findings by Chambon et al. (2013), we found a negative relationship between activity in the left angular gyrus (AG) and agency ratings. Chambon et al., manipulated action fluency by using compatible and incompatible priming and found the AG–agency relationship specifically for high-conflict trials.

Studies directly manipulating participants’ control over (visual) stimuli have found correlates of agency in the more anterior temporo-parietal junction, with higher activity in this region associated with a loss of agency (Farrer and Frith, 2002; Farrer et al., 2003, 2008). The areas reported in these studies are distinct from the more posterior region of the AG described above, but overlap with the TPJ areas we found for the social > nonsocial contrast. Yet, in our study, activity in the TPJ was not related to agency ratings. Possibly, the TPJ areas observed in authorship-related studies, and in our social > nonsocial contrast, are related to the detection of another agent in the environment, rather than to one’s own sense of agency. In authorship-related tasks (Farrer and Frith, 2002; Farrer et al., 2003, 2008), the sense of control over the target stimulus and the likelihood that someone else is involved in the task are strongly negatively correlated and thus cannot be differentiated on a neural level. Our task allowed for the identification of areas related to the presence of a coplayer, but not sense of agency, and vice versa (Fig. 4). Thus, we suggest that anterior TPJ areas monitor the environment for the presence of a coplayer, whereas the posterior AG is related to reduced sense of agency in the face of action selection dysfluency. Our results suggest that this relationship between AG activity and reduced sense of agency is stable across different sources of conflict, be it social context or incompatible response activation (Chambon et al., 2013).

Importantly, although AG and TPJ showed such dissociating patterns of activity for mentalizing and for nonagency, precuneus activity did not show this dissociation. That is, precuneus activity was both increased in the social context and negatively related to sense of agency. Further, precuneus activity did not mediate the social context effect on agency. This presumably reflects a consistent, strong effect of social context on precuneus activity—thus, social context is a reliable cause of increased precuneus activity which, independently of the source of activity increases, is negatively related to sense of agency.

The modulation of precuneus activity by agency ratings was specific for trials with average and high reaction times, but was absent in very fast trials. This might indicate that precuneus activity was related to sense of agency only in trials in which participants had sufficient time to contemplate their action. In line with the above findings that increases in decision-making dysfluency reduce sense of agency over action outcomes (Sidarus et al., 2013, 2017; Sidarus and Haggard, 2016), one straightforward explanation for our current findings is that social cognitive processes interfere with, or at least compete with, task-related action selection and preparation. The relationship between precuneus activity and sense of agency in nonsocial trials may reflect self-referential processes such as adjustments of risk affinity (Lou et al., 2004; Ochsner et al., 2005). Reduced sense of agency over action outcomes might then be the result of metacognitive processes: participants might interpret dysfluency in action-related processes as a signal that the action itself, and its outcomes, are liable to external influences or otherwise error-prone. In conclusion, we propose that precuneus activity in this task is related to assessing relevant hidden attributes of the agents involved in a given context, a process that presumably becomes increasingly complex as the number of other persons involved in the context increases. Such hidden attributes of other agents are relevant for predicting their potential actions and therefore constitute an important source of action-related conflict. This would explain the link between precuneus activation and sense of agency.

### Updated model of social context effects on sense of agency

To summarize, we found that activity in the AG was related to nonagency and does not have any distinct social activation. In contrast, activity in the precuneus was associated with both nonagency and the presence of another possible agent. Based on these findings, we sought to update our model of diffusion of responsibility and, more generally, social context effects on sense of agency (Fig. 6). We propose that sense of agency is prospectively influenced by action-related processing, such as the fluency of action selection and action preparation. Angular gyrus appears to be related to aspects of fluency that are unaffected by social context, such as subjective task difficulty or the availability of a predetermined strategy.

The coplayer’s presence likely evokes a range of cognitive processes: on the one hand, TPJ activity may reflect the mere detection of another agent’s presence in the task environment, which is not related to sense of agency. On the other hand, we suggest that considering the other player’s risk affinity, and thus likelihood to act, and simulating their optimal game strategies, is reflected in increased precuneus activity. Strategic considerations, whether they concern one’s own behavior or that of the other player, compete with the task-related focus, reducing the subjective experience of fluency and ultimately sense of agency. As such, the precuneus sits at an intersection of context-related and action-related cognitive processes, thus linking social contexts to reduced sense of agency. Thus, according to our model, the presence of a coplayer may have similar effects on sense of agency as increasing working memory load or engaging in dual tasks (Hon et al., 2013; Howard et al., 2016; Wen et al., 2016).

It is important to distinguish the processes proposed here from cooperative social settings, in which a sense of joint action may occur and which, if the action is truly collaborative, can result in increased sense of agency (Dewey et al., 2014). Future research needs to show whether mentalizing processes have differential effects on sense of agency depending on the nature of the social interaction.

### MRI results for the outcome phase

Our findings for the outcome presentation phase suggest increased visual attention to outcomes in nonsocial trials, reflected in increased activity in occipital and parietal cortex. This supports our previous finding that outcome monitoring is reduced when the action was performed in a social context (Beyer et al., 2017). However, we found no correlate of increased outcome monitoring in nonsocial trials in prefrontal areas. During social trials, we found increased activity in bilateral TPJ areas and precuneus, suggesting that participants engaged in mentalizing during the outcome phase as well.

Surprisingly, we found no striatal activity in relation to outcome magnitude on a trialwise basis across all task runs. However, activity in the left putamen showed the expected effect—stronger activity for better outcomes—at an uncorrected level for the first task run. This suggests that improved outcome prediction with increasing task experience reduced participants’ neural responsiveness to outcome variability.

### Behavioral findings

Consistent with our previous study (Beyer et al., 2017), participants reported lower sense of agency the more points they lost, and, independently of outcome, lower sense of agency in the social context. This supports our original observation that participants do not strategically blame their coplayer for particularly negative outcomes in social trials (Beyer et al., 2017). Rather, the agency-reducing effect of the other player’s presence is independent of the number of points lost on a given trial. This independence of different influences on sense of agency is consistent with the underlying mechanisms proposed here: whereas the influence of outcome magnitude is by necessity a retrospective effect, we propose that the social context affects sense of agency while the action is being planned and executed, and thus prospectively influences the sense of agency. The highest sense of agency would therefore be associated with individually planned and executed actions that result in positive outcomes.

We did not include reaction times in our model of social context effects on sense of agency, as reaction times were related to outcome magnitude, which was in turn related to sense of agency. Moreover, reaction times also likely depended on individual risk affinity and were directly related to final size of the balloon in a given trial. As such, reaction times showed the opposite relation to sense of agency as might be predicted from the perspective of action fluency, as slower responses were associated with a higher sense of control over action outcomes.

We found no relationship between diffusion of responsibility effects and personality traits of perspective taking or locus of control on the between-subject level. This suggests that reduced sense of agency in social contexts is driven by transient increases in mentalizing processes, rather than by stable personality traits. This suggests important possibilities for counteracting negative effects of social context on individual behavior, e.g., shifting participants’ attention away from the social context toward the immediate task demands should reduce diffusion of responsibility. However, it has to be noted that our sample was primarily drawn from high-functioning student populations. For populations with extreme scores on relevant traits (e.g., samples with callous-unemotional traits or social anxiety), relationships between empathy and diffusion of responsibility may exist.

## Limitations

It should be noted that our interpretation of our findings, i.e., that greater activity in TPJ and precuneus in the social action context may reflect increased mentalizing processes, which in turn interfere with action planning, is based on reverse inference and does not preclude alternative explanations. TPJ and precuneus are strongly associated with mentalizing processes; however, these regions are also activated in other cognitive tasks and, importantly, are part of the default mode network. Further, although our findings support our initial hypotheses, the proposed relationship between mentalizing processes and action planning remains to be clarified in future studies. Therefore, we cannot exclude the possibility that the relationship between social context, precuneus activity, and sense of agency is driven by processes other than decision-making dysfluency. However, as our findings are consistent with a large body of literature on theory of mind and task difficulty in action selection, we are confident that our interpretation is the most straightforward for the current findings.

Further, while we matched visual input between experimental conditions as well as possible, we cannot exclude the possibility that some differences on the neural level may have been related to the presence of the second avatar in social trials. However, as differences between conditions were not focused on visual areas, we do not assume a strong influence of such effects on our results.

## Conclusions

In this fMRI study, when participants acted in a social context, neural activity patterns indicated increased mentalizing. This included increased activity in the precuneus, which was related to reduced sense of agency over action outcomes, independently of social context. Our findings are in line with our theory that in social settings, mentalizing processes interfere with task-related processes, which are necessary for the experience of strong agency over action outcomes. We suggest that processing of action selection and execution is complicated by social cognition, reducing decision-making fluency. For example, in social settings, one needs to consider other agents’ motivation and relevant personality traits, as well as the impact one’s own actions will have on the other’s future behavior. Our findings further suggest distinctive roles for temporo-parietal junction (TPJ) and angular gyrus (AG) in the differentiation between self and others: although TPJ tends to be sensitive to the presence of a coplayer in the task environment, AG is sensitive to sense of agency. Finally, our findings support the proposal that the sense of agency involves the continuous integration of information, from multiple sources, that may become available at various stages of voluntary action—from intention to action to outcome.

**Figure 5.**
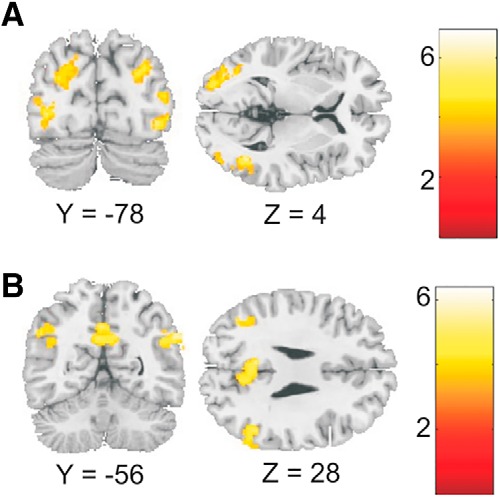
Imaging results for the outcome phase. During nonsocial trials, occipital and parietal cortex showed increased activity (***A***). During social trials, activity was increased in the bilateral temporo-parietal junction and the precuneus (***B***). *n* = 20.

**Figure 6.**
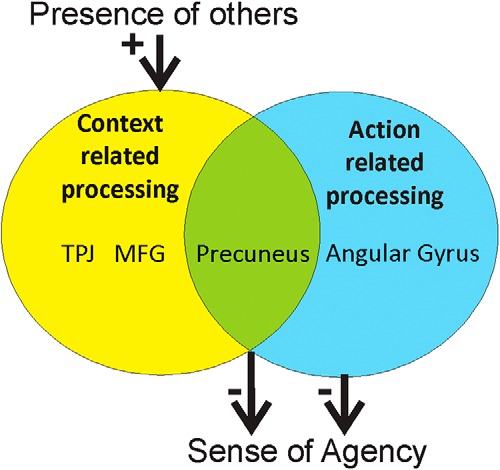
Proposed updated model of social context influences on sense of agency. Our conclusions about influences on sense of agency based on the observed results. Sense of agency is negatively related to neural activity in precuneus and angular gyrus during the action process, likely reflecting action selection dysfluency. Activity in the precuneus is strongly affected by the presence of a coplayer, forming a potential link between social context and loss of subjective agency.

**Table 5.** Results for additional mixed linear model analyses

Factor	β estimate	CI	*t* value	df	*p* value
Model of agency ratings including group factor (MRI/no MRI)					
Social context	0.43	0.18/0.68	3.4	37.1	0.002
Outcome	–0.64	–0.75/–0.52	–10.9	37.4	<0.001
Group	–0.42	–0.91/0.07	–1.7	36.9	0.101
Condition × outcome	0.07	–0.07/0.21	1.0	120.6	0.341
Condition × group	0.21	–0.29/0.70	0.8	37.1	0.418
Outcome × group	0.07	–0.16/0.30	0.6	37.4	0.575
Condition × outcome × group	0.10	–0.18/0.39	0.7	120.6	0.483
Models of precuneus activity by agency ratings and reaction times					
Agency	–0.06	–0.10/–0.02	–2.7	19.8	0.014
RT	–0.04	–0.08/–0.00	–2.0	571.0	0.043
Agency × RT	–0.04	–0.08/–0.01	–2.3	91.4	0.024
Agency effect when using only agency as regressor	–0.06	–0.10/–0.02	–2.9	18.9	0.009
RT time effect when using only RT as regressor	–0.05	–0.09/–0.01	–2.4	643.7	0.015
Model test of precuneus activity by agency ratings for fast, average and slow RTs					
RT group					
–1 SD of mean RT	–0.01	0.03	–0.50	19	0.627
+0 SD of mean RT	–0.05	0.02	–2.70	19	0.014
+1 SD of mean RT	–0.09	0.03	–3.52	19	0.003
Models of angular gyrus activity by agency ratings and reaction times					
Agency	–00.08	–0.16/–0.01	–2.2	37.0	0.035
RT	–00.07	–0.14/–0.01	–2.3	704.1	0.024
Agency × RT	–0.04	–0.10/0.02	–1.2	232.7	0.218
Agency effect when using only agency as regressor	–0.09	–0.17/–0.02	–2.4	37.1	0.020
RT effect when using only RT as regressor	–0.09	–0.15/–0.02	–2.6	407.5	0.009

## Synthesis

Reviewing Editor: Bradley Postle, University of Wisconsin Decisions are customarily a result of the Reviewing Editor and the peer reviewers coming together and discussing their recommendations until a consensus is reached. When revisions are invited, a fact-based synthesis statement explaining their decision and outlining what is needed to prepare a revision will be listed below. The following reviewer(s) agreed to reveal their identity: Keise Izuma.

In response to one of the “minor points” raised by Reviewer 1, you responded as follows:

“We appreciate that more elaborate descriptions of the underlying concepts could be helpful. Unfortunately, due to space restrictions, we were unable to add those to the introduction, but refer to previous articles on the topic, and one using a very similar task (Beyer et al 2017, SCAN) where the rationale is more clearly laid out.” However, as I read through the Introduction, I do not see an explicit reference to ‘mentalizing,’ nor do any of your references to the Beyer paper relate to conceptual points, but rather only to methodological details. Therefore, we ask that you please revise your Introduction to meet the spirit of this comment.

Here are the comments from Reviewer #2

The authors did a great job of addressing the concerns I had previously in general. But, I just have two (relatively minor) comments.

#2e, the authors didn't answer my question. What do you mean by ‘the majority’ exactly? For example, is it 70% or 80%?

#2f, I'm not sure how much space the authors still have, but I strongly believe that they should explain that the reason why they set up their paradigm in a loss frame is because they want to mimic bystander intervention scenarios. This is more important (essential) than “ensuring comparability to the previous study” (Beyer et al., 2017).

#5 Please spell out ‘ICA.”
